# Unique current connecting Southern and Indian Oceans identified from radium distributions

**DOI:** 10.1038/s41598-022-05928-y

**Published:** 2022-02-02

**Authors:** Mutsuo Inoue, Shotaro Hanaki, Hiroaki Kameyama, Yuichiro Kumamoto, Seiya Nagao

**Affiliations:** 1 Low Level Radioactivity Laboratory, Kanazawa University, Wake O-24, Nomi, Ishikawa 923-1224 Japan; 2 Research and Development Center for Global Change, Japan Agency for Marine-Earth Science and Technology, 2-15 Natushima-cho, Yokosuka, Kanagawa 237-0061 Japan

**Keywords:** Environmental sciences, Ocean sciences

## Abstract

We examined the spatial variations in ^226^Ra and ^228^Ra (activities) concentrations from the surface to a depth of 830 m in the Indian and Southern Oceans from December 2019 to January 2020. ^226^Ra concentrations at the surface increased sharply from 30° S to 60° S along a ~ 55° E transect (1.4–2.9 mBq/L), exhibiting small vertical variations, while ^228^Ra decreased southward and became depleted in the Southern Ocean. These distributions indicated the ocean-scale northward lateral transport of ^226^Ra-rich and ^228^Ra-depleted currents originating from the Antarctic Circumpolar Current (ACC). ^226^Ra concentrations indicated that the fractions of the ACC at depths of 0–800 m decreased from 0.95 to 0.14 between 60° S and 30° S. The ACC fractions in the subantarctic western Indian Ocean were higher than those previously reported in the eastern Indian region, indicating preferential transport of the ACC. The fractions obtained were approximately equivalent to those in the western Indian Ocean in the 1970s. This could be attributed to the minimal southward shift of the polar front due to global warming over the last 50 years.

## Introduction

The Antarctic Circumpolar Current (ACC) in the Southern Ocean (> ~ 60° S) is the largest eastward current in the global ocean, surrounding Antarctica. The strong ACC continually connects the Pacific, Atlantic, and Indian Oceans^1–3^. Therefore, this current plays an essential role in the global transport of biomass, nutrients, and pollutants. Additionally, in the subantarctic Indian and Southern Oceans, circulations are unique, with ocean-scale lateral current circulations occurring in the upper, intermediate, deep, and bottom layers^4–6^. The northward lateral currents, originating from the ACC, transport materials from the Southern to Indian Ocean, as well as to the Pacific and Atlantic Oceans, with different transport patterns in each ocean^7–9^. Furthermore, in the Southern Ocean, decadal variability in the Antarctic Polar Front has been recorded, reflecting the influence of climatic changes^10,11^. Therefore, the effects of the recent rapid global warming on ocean circulations are a major concern in marine environments.

Previous studies have investigated the current systems in this area using chemical tracers, such as chlorofluorocarbons (CFCs) and anthropogenic radionuclides^7,12–14^, as well as physical observations^6^. Additionally, natural and soluble radium isotopes such as ^226^Ra (half-life: 1600 years) and ^228^Ra (half-life: 5.75 years) are useful tracers for studying current circulation systems. ^226^Ra in seawater columns is supplied from ^230^Th in the bottom and coastal sediments (and settling particles), owing to its long half-life. The gradients of ^226^Ra (activities) concentrations in water columns have been used to assess the vertical circulation in the global oceans and marginal seas^15^. Spatial variations in ^228^Ra concentrations revealed the lateral circulation of surface seawater affected the coastal and shallow shelf sediments and the subsequent convection^16^. The variations of ^228^Ra concentrations provide implications for the circulation patterns of soluble natural materials and artificial contaminants, such as the radiocesium derived from 2011 Fukushima Dai-ichi Nuclear Power Plant accident^17,18^, in various oceans and seas. Furthermore, the spatial distribution of ^228^Ra concentrations, particularly at the surface, can potentially predict the transport patterns of soluble contaminants.

^226^Ra and ^228^Ra concentrations were primarily examined in the Indian and Southern Oceans from the late 1960s^19–23^, particularly during the expedition of the Geochemical Ocean Sections Study (GEOSECS) conducted from December 1977 to April 1978. The spatial distributions of ^226^Ra and ^228^Ra concentrations showed different features in the subtropical and subantarctic Indian and Southern Oceans, indicating unique and ocean-scale current circulations^24–27^ (and the biological scavenging of ^226^Ra)^28^. This study examined the spatial distributions of ^226^Ra and ^228^Ra concentrations at depths of 10–830 m from the northwestern Indian Ocean to the Southern Ocean and obtained a comprehensive understanding of temporal (over the last 50 years) and ocean-scale spatial variations (between the western and eastern Indian Ocean and the Southern Ocean), incorporating the results from previous studies^19–27^. Furthermore, it clarified ocean-scale current circulations in this area, focusing on the waters connecting the Southern and southern Indian Oceans.

## Results

### Characteristics of current layers

**Figure 1:**
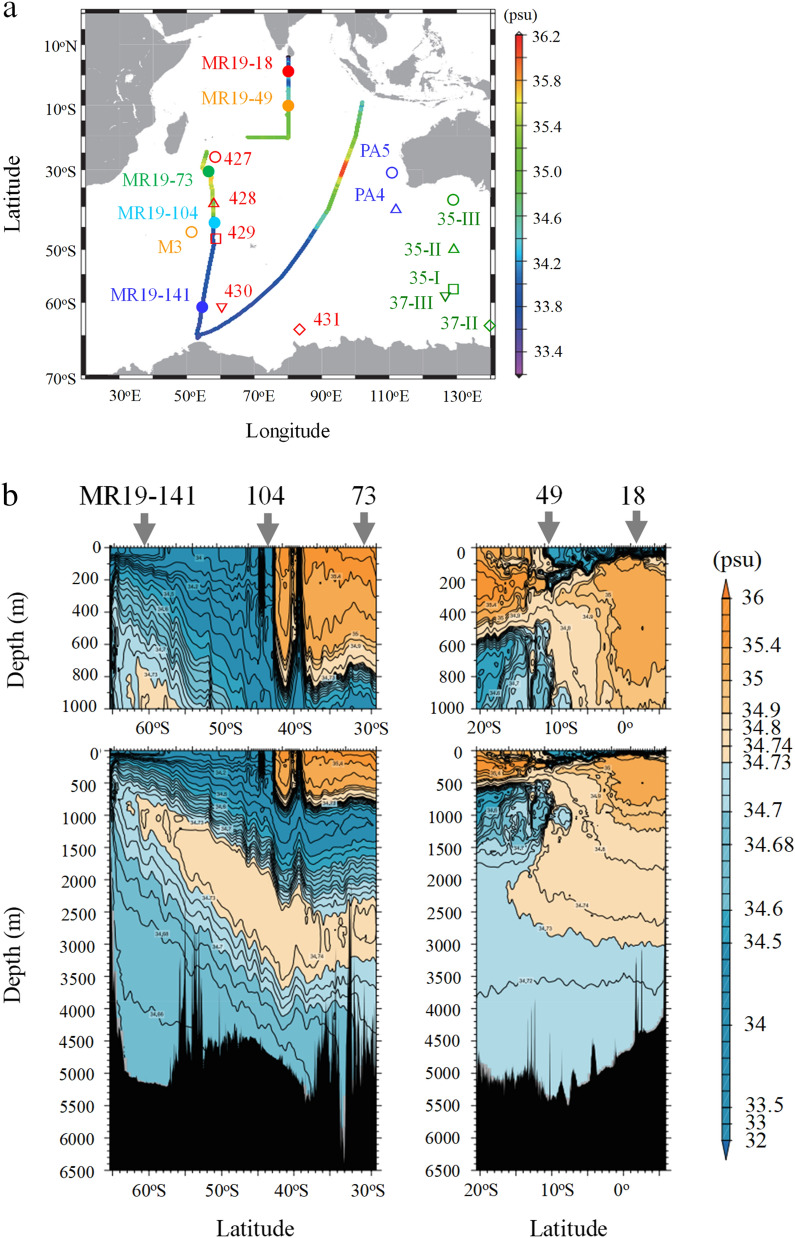

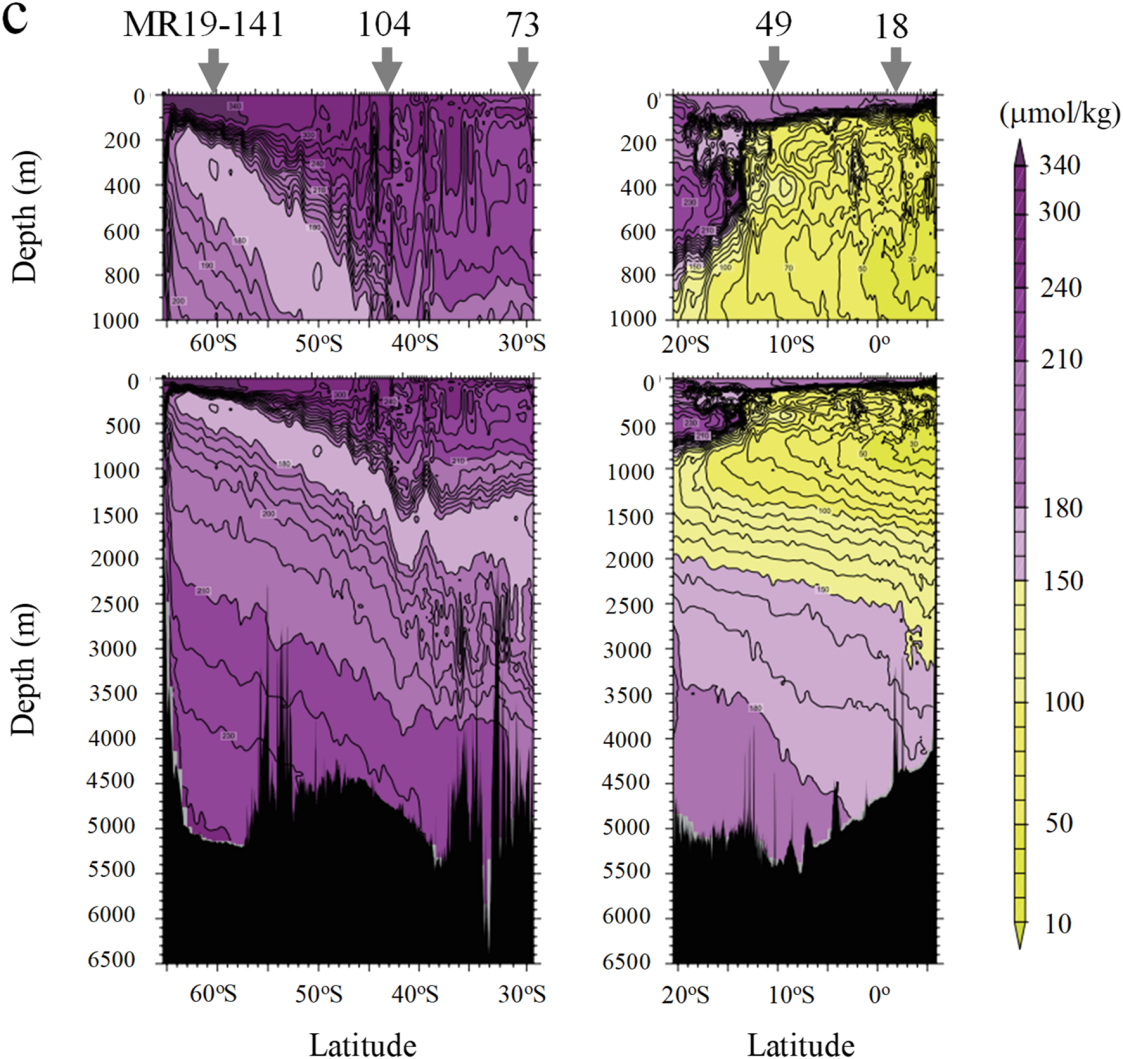
(**a**) Surface salinity along with sampling sites of the MR19-18–141 seawaters in the Indian and Southern Oceans, with key sites from previous studies (sites 35 and 37 in 1968–1969^20^, 427–431 in 1977–1978^24,25^, PA4 and PA5 in 1996^22^, and M3 in 2005^19^), and cross-sectional views of b) salinity and c) dissolved oxygen, along the R/V *Mirai* expedition route^29^. The figures were drawn using the Generic Mapping Tools ver. 4.1.1 (https://www.generic-mapping-tools.org/).

Surface salinity along the expedition route and sampling sites for MR19-18–141 waters are shown in Fig. 1a^29^. @@Cross-sectional observations of salinity and dissolved oxygen (DO) are presented in Fig. 1b,c, respectively. Water columns at sites MR19-18 and -49 contained high-salinity (> 34.5) North Indian Central Water formed by subduction at the subtropical front^4^, which was covered with low-salinity upper-layer waters from the surface to depths of 50–150 m (Fig. 1b). In contrast, low-salinity southward currents (e.g., Antarctic Intermediate Water; AAIW) had a lesser effect on site MR19-49^27^.

The physicochemical characteristics above a depth of ~ 1000 m changed drastically northward of site MR19-104. The salinity of the water columns at sites MR19-104 and -141 was remarkably lower (< 34.7) than that at site MR19-73 (34.5–35.5). Based on the salinity and DO profiles, water columns from depths of > 100–800 m at sites MR19-73 and -104 predominantly comprised the Subantarctic Mode Water (SAMW) and AAIW, which is formed via the convection of Antarctic Surface Water, existing between the upper layer and Upper Circumpolar Deep Water (UCDW)^6,30,31^. The UCDW and Lower Circumpolar Deep Water (LCDW), which may be characterized as the oxygen-minimum and salinity-maximum layers, respectively, spread northward from Antarctica. These layers occupied depths of ~ 200–700 m and ~ 700–1200 m at site MR19-141 and exhibited convective behavior at depths of ~ 1000–2000 m and ~ 2000–3000 m at sites MR19-73 and -104, respectively. The major components of the water columns at sites MR19-73, -104, and -141 (depth of 0–800 m) were the SAMW, AAIW, and UCDW, respectively.

### Surface ^226^Ra and ^228^Ra profiles

**Figure 2:**
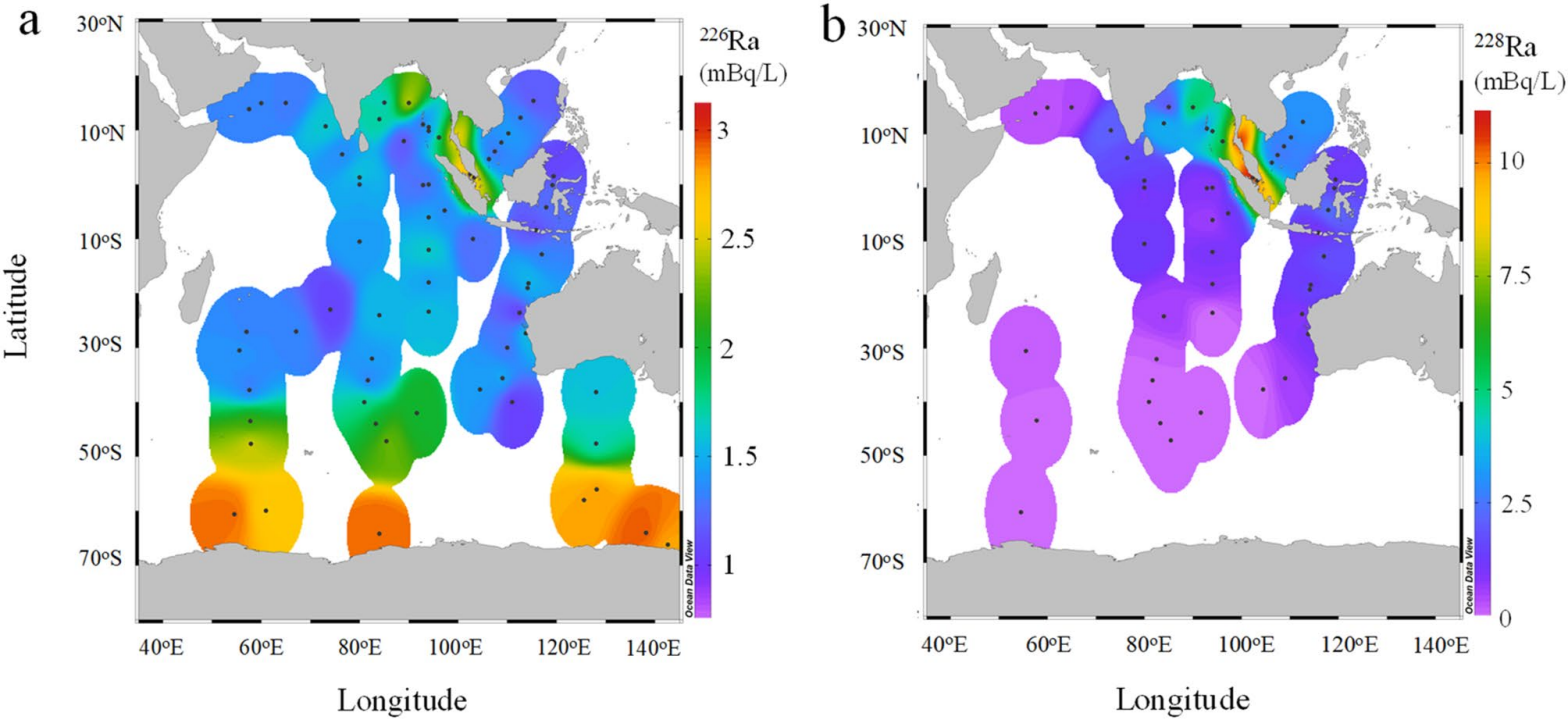
Lateral profile (depth ≤ 10 m) of (**a**) ^226^Ra and (**b**) ^228^Ra concentrations (January–April) in the Indian and Southern Oceans, along with data from previous studies^20–25^. The map in this figure was drawn using Ocean Data View ver. 5.5.1 (http://odv.awi.de).

The lateral (surface) profiles of ^226^Ra and ^228^Ra concentrations in the Indian and Southern Oceans are shown in Fig. 2, along with data from previous studies^20–25^. At the surface, ^226^Ra concentrations along the coasts of Southeast Asia were higher than those in the ambient areas; it decreased towards offshore areas (Fig. 2a). In contrast, the concentrations gradually increased from >  ~ 40° S to the Southern Ocean. Similarly, the ^226^Ra concentrations of surface waters were ~ 1.5 mBq/L at sites MR19-18 and -49; it increased sharply from 1.4 to 2.9 mBq/L between sites MR19-73 and MR19-141.

The ^228^Ra concentrations at the surface along the coasts of Southeast Asia were remarkably high (5–10 mBq/L), overlapping with the high-^226^Ra concentration areas, and decreased sharply towards the offshore, in the equatorial–subtropical area^21–23^ (Fig. 2b). Owing to the short half-life of ^228^Ra, high concentrations of this isotope can be attributed to the mixing of seawaters that have been in contact with the shallow continental shelf and coastal sediments, as observed in the East China Sea^32,33^. In this study, the high ^228^Ra concentrations were predominantly ascribed to the continual supply of ^228^Ra from the coasts of Southeast Asia. Subsequently, ^228^Ra spread to the surrounding areas because of the southwestward monsoon currents, particularly in January^34^. Thus, the ^228^Ra in surface waters at sites MR19-18 and -49 (1.8 and 1.2 mBq/L, respectively) could be attributed to the transport of the southward surface waters; their concentrations further decreased from site MR19-73 to -141 (0.2 to < 0.1 mBq/L).

### Vertical ^226^Ra and ^228^Ra profiles

**Figure 3:**
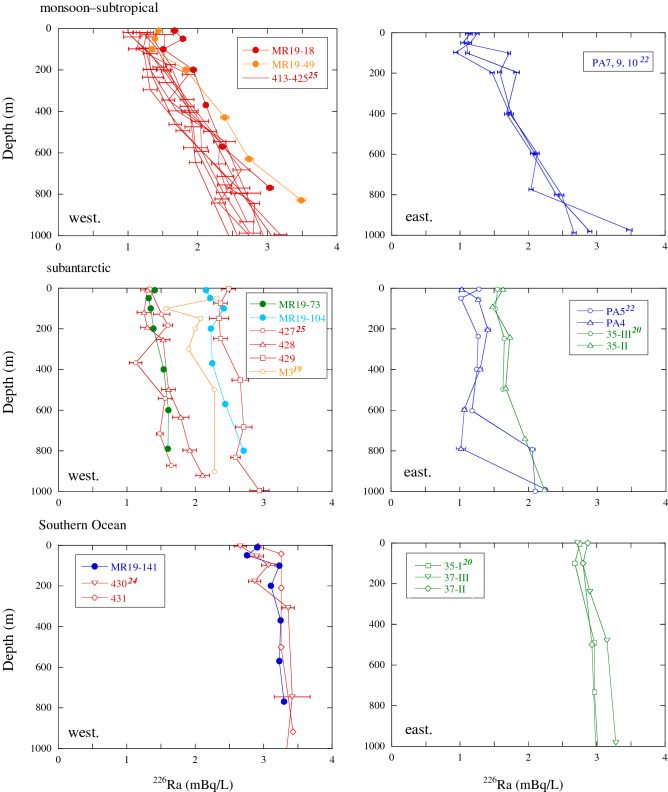
Vertical profiles of ^226^Ra concentrations in the western (west.; < 90° E) and eastern sections (east.; > 90° E) in the monsoon–subtropical (northern side of ~ 20° S) and subantarctic (20–60° S) areas in the Indian Ocean and the Southern Ocean (southern side of ~ 60° S), along with data from previous studies in each area^19,20,22,24,25^.

**Figure 4:**
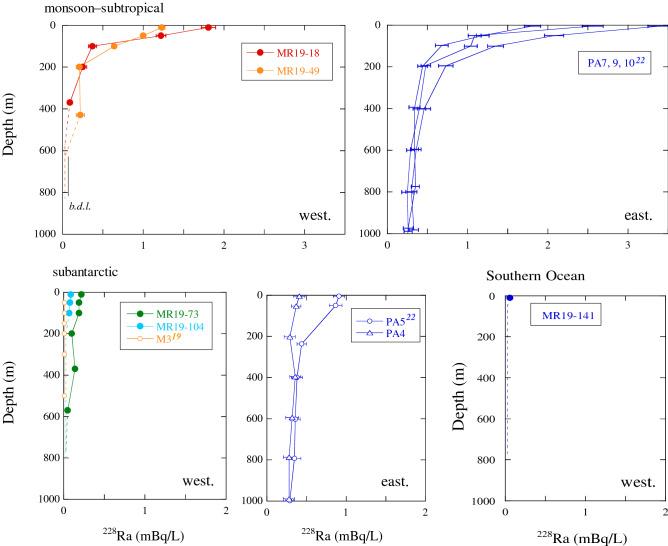
Vertical profiles of ^228^Ra concentrations in the monsoon–subtropical and subantarctic areas in the Indian Ocean and the Southern Ocean, along with data from previous studies in each area^19,22^.

The vertical profiles of the ^226^Ra and ^228^Ra concentrations are shown in Figs. 3 and 4, respectively. The profiles of ^226^Ra concentrations showed three different features in the equatorial–monsoonal and subtropical Indian Ocean (northern side of ~ 20° S), subantarctic Indian Ocean (20–60° S), and Southern Ocean (southern side of ~ 60° S) in both the western (< 90° E) and eastern sections (> 90° E). In the equatorial–subtropical area, the ^226^Ra concentrations gradually increased with depth, in both the western^25^ and eastern Sections ^22^, as well as in the Pacific Ocean in both hemispheres^15^, Sea of Japan^16^, and Bering Sea^35^. Similarly, the ^226^Ra concentrations at sites MR19-18 and -49 increased from 1.5 to 3.5 mBq/L between water depths of 10 and 830 m. At sites MR19-73 and -104 in the subantarctic area, minor vertical variations in ^226^Ra concentrations were observed with depth, particularly below 100 m, relative to those at sites MR19-18 and -49. The concentrations at site MR19-104 were remarkably higher than those at site MR19-73 (2.2–2.7 vs. 1.4–1.6 mBq/L), accompanying the lower salinity at site MR19-104 (Fig. 1b). The ^226^Ra concentrations at sites MR19-73 and -104 were similar to those recorded at nearby sites in 1978 (sites 427–428 and 429, respectively)^25^. In contrast, the ^226^Ra concentrations were higher than those at similar latitudes in the eastern Indian Ocean^20,22^. The ^226^Ra concentrations in the Southern Ocean were considerably higher than those in the subantarctic Indian Ocean at all sites and depths. Although the variations in ^226^Ra concentrations were small in the Southern Ocean, as well as in the western Pacific and eastern Atlantic Sections^28,36^, the concentrations exhibited spatial heterogeneity despite the vigorous lateral circulation of the ACC. The ^226^Ra concentrations at site MR19-141 were approximately equal to those recorded at the nearest sites 430 and 431, which were closer to the concentrations recorded at the same depth in Antarctica (64°11'S) in 1978^24^, and higher than those recorded in the eastern Indian Section^20^. The ^228^Ra concentrations at sites MR19-18 and -49 decreased sharply from the surface to a depth of 100 m, exhibiting stratification at a depth of ~ 100 m (Fig. 4). Such vertical profiles have commonly been observed in the northeastern Indian Ocean^22^ as well as in other oceans^15^ and marginal seas^16^. In addition, the ^228^Ra concentrations in MR19-18 and -49 waters were lower than those in the waters of the eastern Indian Ocean^22^, reflecting a smaller contribution of coastal or shallow shelf waters. Furthermore, the concentrations and vertical gradient of ^228^Ra at site MR19-73 in the subantarctic area were remarkably lower than those at sites MR19-18 and -49; the concentrations below a depth of ~ 100 m were between those observed at sites MR19-49 and -104. The concentrations of ^228^Ra supplied from the Antarctic continental shelf were high along the coast of the Weddell Sea (~ 4 mBq/L at 20 m depth) and decreased sharply offshore^37^. The ^228^Ra concentrations in the waters from site MR19-141 in the Southern Ocean area were the lowest in the study area, at all depths, as observed at the same latitude in the Weddell Sea (< 0.1 mBq/L).

### ^226^Ra versus ^228^Ra concentrations

**Figure 5:**
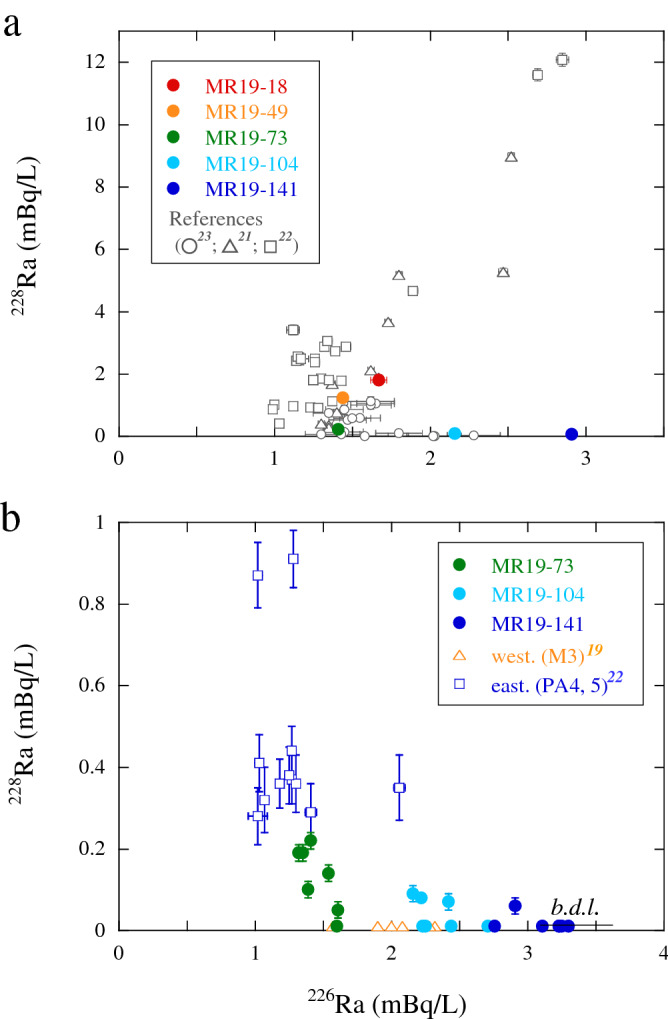
^226^Ra versus ^228^Ra concentrations of waters from (**a**) the surface (depth ≤ 10 m) and (**b**) surface to a depth of 800 m, along with data from previous studies in the Indian and Southern Oceans^19,21–23^.

Figure 5 shows the concentrations of ^226^Ra plotted against those of ^228^Ra for MR19 waters, along with previous data from the Indian and Southern Ocean^19,21–23^. The seasonal variations in ^228^Ra and ^226^Ra concentrations, particularly in the northeastern Indian Ocean, were unclear (e.g., due to monsoon currents)^34^; however, the concentrations recorded at the surface from January to April exhibited a positive correlation (Fig. 5a). This predominantly reflects the supply of ^226^Ra and ^228^Ra from shallow shelf and coastal sediments along the coasts of Southeast Asia. However, surface waters with high ^226^Ra concentrations at sites MR19-73, -104, and -141 and at a few sites in the subantarctic Indian Ocean^23^ exhibited the minimum ^228^Ra concentrations.

The ^226^Ra concentrations (< 2 mBq/L) of waters at site MR19-73, including the eastern area (sites PA4 and PA5)^22^, above depths of 800 m exhibited a negative correlation with ^228^Ra concentrations (Fig. 5b). However, the waters in the eastern area, particularly at depths of 5 and 50 m at site PA5, reflect the contribution of ^228^Ra-rich surface layer waters (Fig. 4). The ^228^Ra concentrations in samples from sites MR19-104 and -141 with high ^226^Ra concentrations (> 2 mBq/L), as well as a site in the western area (~ 0.01 mBq/L at M3)^19^, were below the detection limit (< 0.03 mBq/L). Additionally, low ^228^Ra concentrations at site MR19-141 indicated that the contribution of waters affected by the ^228^Ra-rich continental slopes and coastal sediments along Antarctica was minimal. Additionally, the transport of waters from the Antarctic continental shelf to the offshore area was slow (1.5 years)^37^. Based on the spatial distributions of ^228^Ra and ^226^Ra concentrations (Figs. 3 and 4), water columns at sites MR19-18 and -49 were similar to those typically observed in open oceans^15^ and marginal seas^16,35^. In contrast, the spatial distributions of ^228^Ra and ^226^Ra concentrations at sites MR19-73, -104, and -141 could be explained based on different current circulation systems.

## Discussion

**Figure 6:**
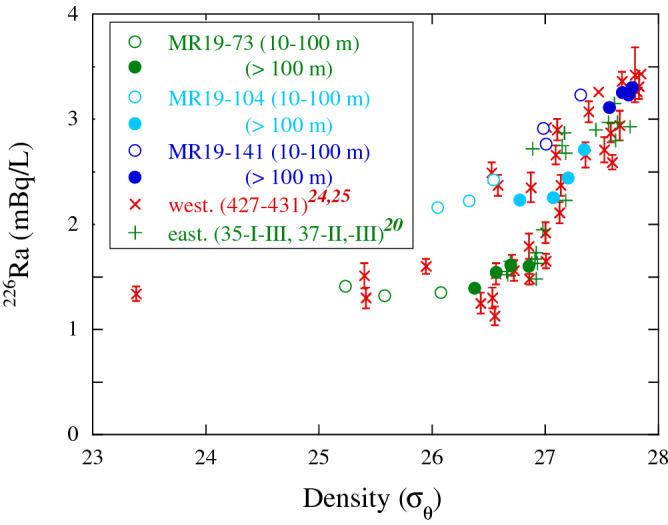
^226^Ra concentrations versus potential density of waters from the surface to a depth of 800 m, along with data from previous studies in the Indian and Southern Oceans^20,24,25^.

Figure 6 shows the ^226^Ra concentrations of MR19 waters plotted against the potential density, along with previously reported data form the Indian and Southern Oceans^20,24,25^.

The ^226^Ra concentrations of the upper-layer waters (10–100 m depth) at sites MR19-73, -104, and -141 likely exhibit a positive correlation with density, showing a less-steep slope (n = 9; r^2^ = 0.87), than those at a depth > 100 m. This indicates the mixing of the southward upper-layer currents, such as the subantarctic surface water (~ 1.5 mBq/L for ^226^Ra and ~ 25σ_θ_). Therefore, it is inferred that the intrusion of the southward upper currents shifted the density and ^226^Ra concentrations of water at depths of 0–100 m to lower sides.

Moreover, the ^226^Ra concentrations in MR19 waters, particularly at depths of > 100–800 m at sites MR19-73, -104, and -141, were positively correlated with density (n = 12; r^2^ = 0.94). The ^226^Ra concentrations gradually increased with depth in the bottom waters in the subtropical area, reflecting the supply of ^226^Ra from the bottom sediments^25^; however, the concentrations were approximately constant below 1000 m in the subantarctic and Southern Oceans, even in the deep and bottom waters^20,24^. Based on the salinity and DO features (Fig. 1b,c), the water columns at sites MR19-73 and -104 are strongly stratified into the SAMW (26.5–26.8σ_θ_) and AAIW (27.0–27.3σ_θ_), respectively, which disrupts the intense vertical mixing between these layers. Additionally, the ^226^Ra concentrations of MR19 waters were two orders of magnitude higher than those of the reactive and parent ^230^Th^38^; this indicates that ^226^Ra is largely supplied from bottom and coastal sediments. Therefore, high ^226^Ra concentrations and small vertical variations at site MR19-104 can be explained by the lateral transport of ^226^Ra-rich waters rather than the upwelling of ^226^Ra from the LCDW. The ^226^Ra-rich waters at MR19-141 in the Southern Ocean are mainly from UCDW (~ 27.6σ_θ_), which plays a key role in increasing the ^226^Ra concentrations of water columns at sites MR19-104 and -73.

**Figure 7:**
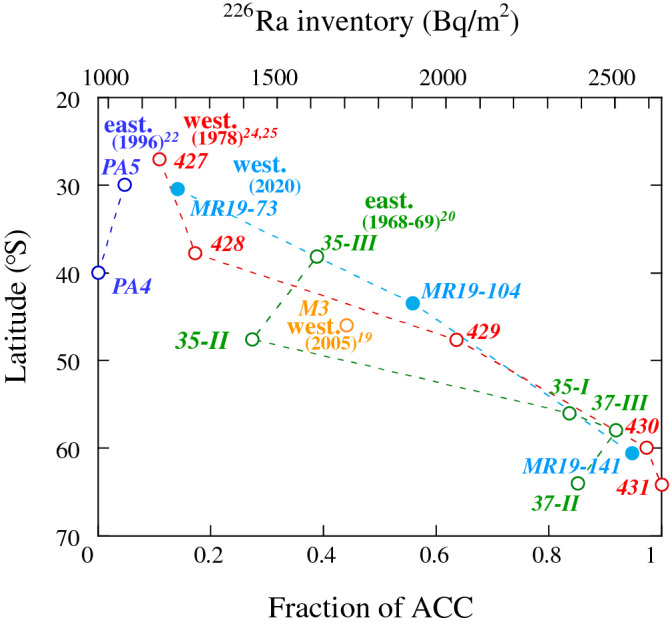
Inventories of ^226^Ra concentrations (depth of 0–800 m) and fractions of the ACC (1.0 at site 431) versus latitude, compared with data from previous studies recorded in the western^19,24,25^ and eastern Sections^20,22^ of the subantarctic Indian and Southern Oceans over the last 50 years.

Water column inventories (depth of 0–800 m) of ^226^Ra concentrations in the subantarctic Indian and Southern Oceans are plotted against latitude in Fig. 7 and compared with the values from previous studies^19,20,22,24,25^. The inventories increased sharply from site MR19-73 (1205 Bq/m^2^) to MR19-141 (2550 Bq/m^2^) via site MR19-104 (1900 Bq/m^2^). Inventories at sites MR19-73 and -104 in the subarctic area were evidently similar to those obtained at nearby sites in 1978 (sites 427–428 and 429, respectively)^25^. In contrast, the inventories decreased from the western Indian Ocean to offshore western Australia in the eastern Indian Ocean (850 Bq/m^2^ at PA4 and 940 Bq/m^2^ at PA5)^22^. Combined with the high ^228^Ra concentrations (Figs. 2b and 4), the low inventories of ^226^Ra at sites PA4 and PA5 can be attributed to the intrusion of southward currents with high ^228^Ra and low ^226^Ra concentrations (e.g., the Leeuwin Current)^39^. Furthermore, the inventory yielded a higher value at site MR19-104 than that at similar latitudes in southern Australia (1426 and 1616 Bq/m^2^ at sites EL35-II and -III, respectively)^20^. Such wide variations in the ^226^Ra inventories, particularly from 30° S to 50° S, indicate different transport patterns of ACC from the Southern Ocean to the western and eastern Indian Oceans.

The salinity at site MR19-141 in the Southern Ocean was at the same level as that at site 430, except for a lower value above a depth of ~ 50 m^40^, indicating a larger contribution from Antarctic water. High-density (> 27σ_θ_) and high-^226^Ra features in the upper-layer waters at site MR19-141, which are equivalent to those at sites 430 and 431 (Fig. 6), indicate less mixing of low-density and low-^226^Ra currents, relative to that in the eastern area. High ^226^Ra concentrations at site MR19-141 could have been caused by a greater contribution from the ^226^Ra-rich ACC.

The ^226^Ra inventory at site 431 (2638 Bq/m^2^) exhibited the highest value in this study area^24^. Conversely, the ^226^Ra concentrations above a depth of 800 m at site PA4 were equivalent to the lowest values recorded in the Indian Ocean, such as the upper layer in the northern Indian Ocean (Fig. 3). Therefore, we considered the water columns from depths of 0 to 800 m at sites 431 and PA4 (970 Bq/m^2^) as the end-members in the ACC and the water from the northern Indian Ocean, respectively. Consequently, the fractions of the ACC (i.e., column waters at site 431) were estimated as 0.95 at MR19-141, 0.56 at MR19-104, 0.14 at MR19-73, and 0.44 at site M3, based on their inventories (Fig. 7). The fractions at sites EL35-II and -III in the eastern Indian Ocean (0.27 and 0.39, respectively) correspond to ~ 60% of that at site MR19-104, despite their similar latitude. Higher inventories in the western subantarctic Indian Ocean could be explained by the direct inflow of the ACC, compared to that in the eastern area, where the ACC has longer pathways (< 10 and < 30 years to the west and east, respectively, based on CFC age)^7,31^, and by the large-scale intrusion of southward currents observed offshore of western Australia^22^.

Decadal variations in seawater temperature have recently been recorded in and around the polar front, reflecting changes in global climate^11^. Anomalous warming below depths of 200 m is accompanied by density anomalies in the Southern Ocean^41,42^. The fraction at site MR19-141 was comparable to that at the closest site 430 (2593 Bq/m^2^, 0.97). Additionally, the ACC fractions at sites MR19-104 and -73 exhibited similar values to those observed at sites 429 (0.64) and 428–427 (0.17–0.11) in 1978^25^. This can be primarily attributed to the minimal effects of the southward shift of the polar front^10^ and/or the short-term (e.g., annual) and local variations in the circulation of the ACC-dominated water^43^ and eddy mixing^44^. Compared to earlier reports, our study of water currents using ^226^Ra and ^228^Ra did not indicate any significant decrease in the contribution of the ACC that could be attributed to the southward shift of the polar front due to global warming since the 1970s.

## Methods

### Seawater samples and experiments

A total of 35 seawater samples (~ 40 L each) were collected at depths of 10–830 m from five water columns in the Indian and Southern Oceans using a conductivity–temperature–depth rosette with 36 Niskin-X bottles (12 L) during the R/V *Mirai* expedition from December 2019 to January 2020 (MR19-04 expedition) (Fig. 1a). All water samples were unfiltered. The experimental procedures employed to collect Ra in seawater samples have been previously described^45^. After adjusting the pH to ~ 1 using concentrated HNO_3_, a minimally Ra-contaminated Ba carrier (960 mg) was added to a ~ 40 L aliquot of each seawater sample, and BaSO_4_ was precipitated with the Ra isotopes. The chemical yields of the Ra isotopes were 93–100%, based on the yields of the BaSO_4_ fractions.

Low-background γ-spectrometry was performed on all BaSO_4_ samples using high-purity Ge-detectors located in the Ogoya Underground Laboratory, Japan^46^, over 3–5 counting days. The ^226^Ra concentrations were evaluated from the γ-ray peaks of ^214^Pb (295 and 352 keV) after reaching radioactive equilibrium (> 3 weeks after sample compression); they were calibrated from the peak ratios of mock-up samples with almost the same chemical composition as the water samples, including uranium standards issued by the New Brunswick Laboratory, USA (NBL-42-1). In addition, ^228^Ra concentrations were characterized from ^228^Ac (338 and 911 keV), based on the detection efficiency curve obtained from the mock-up samples.

Under the analytical conditions employed in this study, the minimum amount of ^228^Ra that could be determined in a water sample was ~ 1.5 mBq. This corresponded to a detection limit of ~ 0.03 mBq/L when using ~ 40 L of sampled seawater. Based on the standard deviation, the analytical precision was 1–3% and 3–30% for ^226^Ra and ^228^Ra, respectively. Both the ^228^Ra and ^226^Ra concentrations in this study were decay-corrected to the sampling date.
